# Data mining EEG signals in depression for their diagnostic value

**DOI:** 10.1186/s12911-015-0227-6

**Published:** 2015-12-23

**Authors:** Mahdi Mohammadi, Fadwa Al-Azab, Bijan Raahemi, Gregory Richards, Natalia Jaworska, Dylan Smith, Sara de la Salle, Pierre Blier, Verner Knott

**Affiliations:** 1 Knowledge Discovery and Data mining Lab (KDD), University of Ottawa, Ottawa, ON Canada; 2 Department of Psychiatry, McGill University, Montreal, QC Canada; 3 Department of Cellular and Molecular Medicine, University of Ottawa, Ottawa, ON Canada; 4 School of Psychology, University of Ottawa, Ottawa, ON Canada; 5 Institute of Mental Health Research at the Royal Ottawa Mental Health Care Centre, University of Ottawa, 1145 Carling Avenue, Ottawa, ON K1Z 7 K4 Canada

**Keywords:** Depression, EEG, Genetic algorithm, Linear discriminant analysis, Decision tree, Sensitivity, Specificity

## Abstract

**Background:**

Quantitative electroencephalogram (EEG) is one neuroimaging technique that has been shown to differentiate patients with major depressive disorder (MDD) and non-depressed healthy volunteers (HV) at the group-level, but its diagnostic potential for detecting differences at the individual level has yet to be realized. Quantitative EEGs produce complex data sets derived from digitally analyzed electrical activity at different frequency bands, at multiple electrode locations, and under different vigilance (eyes open vs. closed) states, resulting in potential feature patterns which may be diagnostically useful, but detectable only with advanced mathematical models.

**Methods:**

This paper uses a data mining methodology for classifying EEGs of 53 MDD patients and 43 HVs. This included: (a) pre-processing the data, including cleaning and normalization, applying Linear Discriminant Analysis (LDA) to map the features into a new feature space; and applying Genetic Algorithm (GA) to identify the most significant features; (b) building predictive models using the Decision Tree (DT) algorithm to discover rules and hidden patterns based on the reduced and mapped features; and (c) evaluating the models based on the accuracy and false positive values on the EEG data of MDD and HV participants. Two categories of experiments were performed. The first experiment analyzed each frequency band individually, while the second experiment analyzed the bands together.

**Results:**

Application of LDA and GA markedly reduced the total number of utilized features by ≥ 50 % and, with all frequency bands analyzed together, the model showed average classification accuracy (MDD vs. HV) of 80 %. The best results from model testing with additional test EEG recordings from 9 MDD patients and 35 HV individuals demonstrated an accuracy of 80 % and showed an average sensitivity of 70 %, a specificity of 76 %, and a positive (PPV) and negative predictive value (NPV) of 74 and 75 %, respectively.

**Conclusions:**

These initial findings suggest that the proposed automated EEG analytical approach could be a useful adjunctive diagnostic approach in clinical practice.

**Electronic supplementary material:**

The online version of this article (doi:10.1186/s12911-015-0227-6) contains supplementary material, which is available to authorized users.

## Background

Depression, a common psychiatric disorder with a lifetime prevalence of ~ 20 % in the general population, is associated with high rates of disability, impaired psychosocial functioning and decreased life satisfaction [1]. Early recognition and accurate diagnosis of depression are essential criteria for optimizing treatment selection and improving outcomes, thus reducing the economic and psychosocial burdens resulting from hospitalization, lost work productivity and suicide [2–4]. Guided by established classification criteria (DSM-5) [5], the diagnosis of psychiatric disorders including depression relies solely on inferences based on self-reported information and observed behaviour. Identifying people with established depression does not usually present as a clinical challenge with standard clinical instruments but the potential for ambiguity, bias and low reliability of a diagnosis of depression based on clinical descriptions can be compounded by the heterogeneous nature of the disorder. There are a number of DSM-5 defined depressive disorders (e.g. major depressive disorder [MDD], dysthymia, depressive disorder not otherwise specified [NOS]) and, for unipolar MDD, there are symptom based subtypes (e.g. melancholic, psychotic and atypical depression); symptoms can also vary by gender, age and even race [6].

Defined as objective biological measures indicating the state of a normal biologic process, pathogenic process, or pharmacological response to a therapeutic intervention [7], biomarker use for diagnostics has become standard in day-to-day practice in medicine (e.g. cardiology, oncology) but there are no accepted biomarkers for MDD or other psychiatric disorders. Recent progress has provided evidence that psychiatric disorders are brain disorders characterized by abnormalities in the structure, function and neurochemistry in distributed neural networks [8]. Neuroimaging, which allows for *in vivo* access to these brain circuits, has increased our understanding of the pathophysiology of these disorders [9, 10] and is a leading candidate for the development of clinical biomarkers with potential use for diagnosis, prognosis and treatment of depression [11–16].

For biomarkers to be diagnostically useful, they need to be reliable and reproducible, providing sufficiently high levels of sensitivity and specificity in the detection and correct classification of distinct disorders [17]. Furthermore, for routine use in clinical practice, they should be inexpensive, noninvasive and easily accessible [17]. Compared to some other proposed brain imaging biomarkers derived from functional magnetic resonance imaging (fMRI), positron emission tomography (PET), and magnetic resonance spectroscopy (MRS), quantitative measurement of brain electrical signals taken from the scalp-recorded electroencephalogram (EEG) is a neuroimaging technique with clear practical advantages as it does not involve invasive procedures, is widely available, easy to administer, well tolerated, and has a relatively low cost [18]. In addition to its growing potential as a biomarker in the therapeutic drug development process [19, 20] and in predicting antidepressant treatment response [21–25], power spectral measures of resting state EEG oscillatory activity in different frequency bands (delta [<4 Hz], theta [~4–8 Hz], alpha [~8–12 Hz], beta [~12–30 Hz) have been shown to distinguish between depressed patients and healthy controls [26- 28]. However, EEG biomarkers/biosignatures characterizing brain abnormalities in depressed patients tend to be limited to group-level comparisons. Although they are informative in elucidating the neuropathophysiology of depression, investigations have not systematically examined whether or not these EEG measurements can be useful, at the individual level, in diagnosing whether a given subject is or is not depressed.

Studies focusing on individual-level neuroimaging data analyses are necessary if this approach is to be clinically useful [16] but the inherent complexity of the data and its analyses continues to be an obstacle [10]. Recent advances in EEG acquisition (high density systems) and processing has added to this complexity but this growth has been paralleled by the increased availability of machine learning methods. Unlike conventional analyses, machine learning classifiers are designed to deal with multivariate inputs — treating the EEG measures as patterns rather than considering each measure in isolation [16, 29, 30]. To date, the limited number of machine learning studies on resting state EEG in depression have used varying classification algorithms and have been found to classify MDD patients and healthy controls with an overall accuracy ranging between 60–90 % [31–35].

Despite their promise as a supplementary, computer-aided diagnostic approach, these analytic methods have not clearly delineated the contributing role of oscillatory activity in each frequency band and/or brain region to the machine learning classifiers. Further, they have not yet examined the role of vigilance states (e.g. eyes open vs. eyes closed) or recording montages (e.g. unipolar vs. bipolar EEG recordings). It is also unclear from the existing machine learning EEG studies if classification accuracy is different when analyzing data from each frequency band compared to when data from all bands are analyzed together.

EEG is sensitive to a continuum of states ranging from stress states, alertness to resting state, and sleep, and various regions of the brain do not emit the same oscillatory activity simultaneously. During the normal state of wakefulness with eyes open fast frequency (beta) oscillations are dominant in central-frontal scalp areas. During relaxation recorded in an eyes-closed resting condition, alpha activity in the EEG is dominant in posterior scalp regions and is markedly diminished when individuals open their eyes, perhaps reflecting widespread communication of cortical and thalamo-cortical interactions to aid information processing of visual input [36, 37].

Several difference recording reference electrode placements are mentioned in the literature. The choice of reference may produce topographic distortion in oscillatory signals if a relatively electrically neutral site is not employed. Referencing to linked mastoids/earlobes and vertex scalp (Cz) are predominant in the depression EEG literature and may account for differences across studies as each technique has its own set of advantages and disadvantages. Linking reference electrodes from two earlobes or mastoids reduces the likelihood of artificially inflating activity in one hemisphere but this method may drift away “effective” reference from the midline plane if electoral resistance at each reference electrode differs [38]. Cz reference is advantageous when it is located in the middle among active electrodes, however for closer points it makes poor resolution.

In this study, multi-feature data mining methodologies were used to classify MDD patients and non-depressed individuals using EEG data in six frequency bands derived from 28 scalp sites during both eyes-open and eyes-closed resting states, and computed with mastoid-based unipolar (measuring the difference between EEG signals at the scalp and a neutral non-scalp signal) and Cz-based bipolar (measuring the EEG difference between pairs of EEG scalp signals) referenced recordings. The aim was to assess whether these analytical approaches to EEG may provide an objective complementary tool to MDD diagnosis.

## Methods

### Project participants

A sample of 53 adults with a primary diagnosis of MDD and 43 age matched healthy volunteer (HV) adults participated in this study. MDD diagnoses were psychiatrist-confirmed using the Structured Clinical Interview for DSM (Diagnostic and Statistical Manual of Mental Disorders) IV-TR Diagnoses, Axis I, Patient Version (SCID-IV-I/P) [39]. The majority of patients had previous MDD episodes. The Montgomery-Asberg Depression Rating Scale (MADRS) [40] was used to assess symptom severity. All patients scored ≥22 (moderate depression) on the MADRS, and the mean being 30.8 (standard deviation [S.D.] ± 5.2). Notable study exclusion criteria included: Bipolar Disorder (BP-I/II or NOS), a history of psychosis, current (<6 months) drug/alcohol abuse or dependence, history of seizures or known increased seizure risk, and any unstable medical condition. Patients presenting with a significant risk for suicide were excluded, but those with a secondary diagnosis of some anxiety disorder were included (*N* = 33: no anxiety comorbidity; *N* = 12: sub-threshold anxiety; *N* = 8; secondary diagnosis of some form of anxiety). Appropriate drug washout periods were employed prior to testing for any previously medicated patients; all patients were medication-free at the time of testing.

HVs were assessed with the non-patient version of the SCID (SCID-IV-I/NP) and were excluded if they exhibited a psychiatric, neurological (seizures, brain trauma) or alcohol/drug abuse or dependence history. They were included only if they scored ≤ 13 on the Beck Depression Inventory-II (BDI-II) [41] and had no psychiatric history in first-degree relatives (Family Interview for Genetic Studies [FIGS]) [42].

This study was approved by the Royal Ottawa Health Care Group and the University of Ottawa Social Sciences and Humanities Research Ethics Boards. Written informed consent was obtained from all participants; each was compensated $30.00 CAN per testing session.

### EEG acquisition

**Fig. 1:**
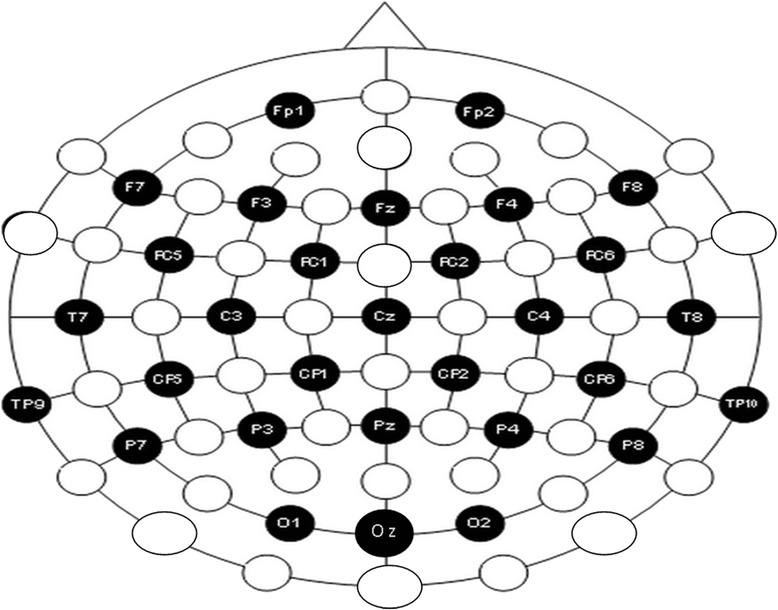
Electrode positions

Participants were required to abstain from caffeine and nicotine (minimum of 3 hrs) and alcohol and drugs (beginning at midnight) prior to their test session. Upon arrival at the laboratory, they were seated in a sound- and light-attenuated chamber, where EEG recordings were obtained during 3 min vigilance-controlled eyes-closed (EC) and 3 min eyes-open (EO) resting conditions (counter-balanced). EEG was recorded (sampling rate 500 Hz) in reference to activity from electronically linked mastoids and using a cap system with 28 Ag/AgCl scalp electrodes (EasyCap, Herrsching-Breitbrunn, Germany) positioned on the scalp according to the 10–10 system [43] (Fig. 1). Electrodes placed on the external canthi and on the supra- and sub-orbital ridges recorded electrooculographic (EOG) activity, and an electrode at AF_z_served as the ground. Electrode impedances were maintained at ≤ 5 kΩ and electrical signals were recorded with amplifier bandpass filter settings of 0.1–80 Hz using a BrainVision Quickamp amplifier and BrainVision Recorder Software (BrainVision, Richardson, TX, USA).

### EEG processing

EEG data was processed off-line using BrainVision Analyzer Software (BrainVision, Richardson, TX, USA). Signals were referenced with electronically linked mastoid electrodes (TP_9/10_) or a scalp vertex (Cz) electrode to yield two data sets for each of the EC and EO recordings. For both referenced recordings, signals were filtered (0.1–30 Hz), ocular corrected [44], and segmented into 2 s epochs (50 % overlap). Subsequent automatic artifact rejection was used to exclude epochs with activity exceeding +/− 75 μV. The remaining epochs were visually inspected for additional artifacts and faulty channels. For each of EC and EO data sets (linked mastoid and Cz references), >100 s artifact-free signals from each of the 28 electrodes (TP_9/10_ was not used in the analyses) were subjected to a Fast Fourier Transform (FFT) algorithm (Hanning window with 5 % cosine taper) for computation of both absolute and ln-transformed power (μV^2^) in delta (1–4 Hz), theta (4–8 Hz), alpha_1_, (8–10.5 Hz), alpha_2_ (10.5–13 Hz), alpha total (8–13 Hz) and beta (13–30 Hz) frequency bands.

Table 1 provides an overview of the raw data variables collected in 53 depressed patients (MDD) and 43 healthy volunteers (HV) before data pre-processing. Power values for the four main bands were obtained for each of the 28 electrodes using two reference montages (mastoids and Cz) during the eyes-open (EO) and eyes-closed (EC) conditions. As such, a total of 12 EEG datasets existed.

**Table 1:** Data description

References	Bands	Condition	Sites	Frequencies	Samples	
Mastoids	Alpha	EO	Fp1, Fp2, F3, F4, C3, C4, P3, P4, O1, O2, F7, F8, T7, T8, P7, P8, Fz, Cz, Pz, Oz, Fc1, Fc2, Cp1, Cp2, Fc5, Fc6, Cp5, Cp6	(8–10.5 Hz)	HV	43
		EC		(10.5–13 Hz)	MDD	53
	Beta	EO		(13–30 Hz)	HV	43
		EC			MDD	53
	Theta	EO		(4–8 Hz)	HV	43
		EC			MDD	53
	Delta	EO		(1–4 Hz)	HV	43
		EC			MDD	53
Cz	Alpha	EO	Fp1, Fp2, F3, F4, C3, C4, P3, P4, O1, O2, F7, F8, T7, T8, P7, P8, Fz, Cz, Pz, Oz, Fc1, Fc2, Cp1, Cp2, Fc5, Fc6, Cp5, Cp6	(8–10.5 Hz)	HV	43
		EC		(10.5–13 Hz)	MDD	53
	Beta	EO		(8–10.5 Hz)	HV	43
		EC		(13–30 Hz)	MDD	53
	Theta	EO		(4–8 Hz)	HV	43
		EC			MDD	53
	Delta	EO		(1–4 Hz)	HV	43
		EC			MDD	43

### Data mining

**Fig. 2:**
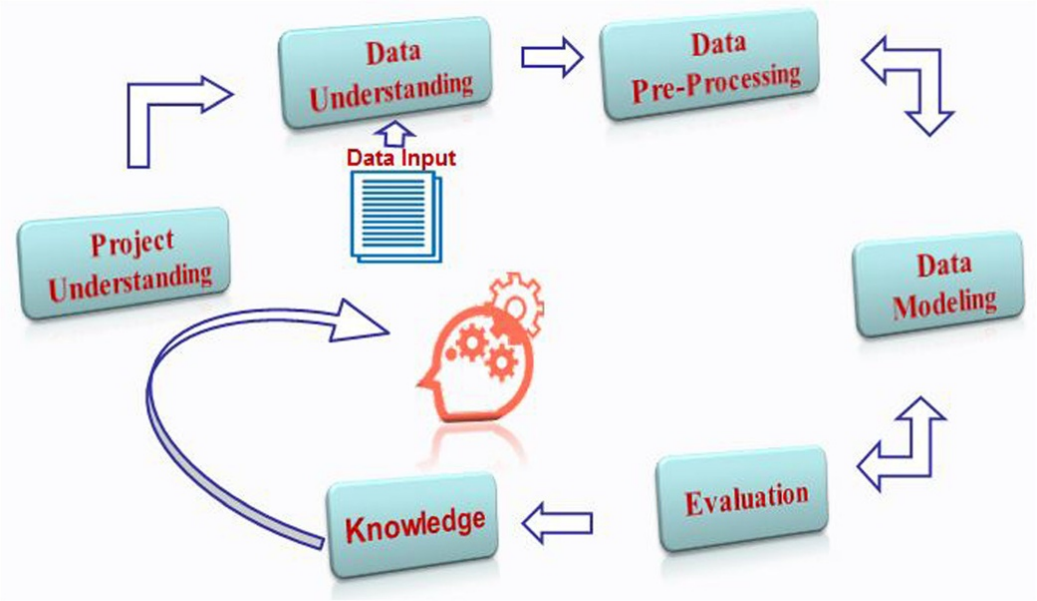
Data mining methodology

The *Data Mining Methodology* was chosen to provide an outline about the study’s life cycle to tackle the stated problem and to describe the processes, techniques and models involved in achieving the study’s goals. The methodology consists of six phases (Fig. 2), starting with the initial phase of understanding the project (Introduction), followed by data understanding (EEG Acquisition/Processing), data pre-processing, data modeling, evaluation and ending up with the deployment (knowledge discovery) [45]. The detailed description of each phase applied in this study is outlined below.

#### Data pre-processing

The data pre-processing phase includes all the tasks that are performed to construct and prepare the raw data into final datasets in order to be fed into the data modeling phase [45]. The pre-processing phase consisted of four main steps which are listed as follows:

##### Data cleaning

The cleaning phase was performed in Microsoft Excel. Once the datasets were structured and examined for missing values, a small experiment was implemented to determine whether absolute or log power performed better. The results showed that the absolute power values yielded better accuracy compared with the log power values.

##### Data transformation

Data transformation was performed by applying a normalization technique to make all the data values fall within a small range (0–1) to enable the efficient performance of the predictive model (classifier). The employed normalization technique is *min-max normalization*, which performs a linear transformation on the original data by setting the min value to 0 and max value to 1. “*The minimum and maximum values are represented in the formula (**1**) with min*_*A*_*and max*_*A*_*of an attribute A. Min-max normalization maps a value, v*_*i*_*of A to v*_*i*_’ *in the range* [*new*_*max*_*A*_, *new*_*min*_*A*_]*”* [46].

1 $$ {\boldsymbol{v}}^{\boldsymbol{\hbox{'}}}=\frac{\boldsymbol{v} - \boldsymbol{m}\boldsymbol{i}{\boldsymbol{n}}_{\boldsymbol{A}}}{\boldsymbol{ma}{\boldsymbol{x}}_{\boldsymbol{A}} - \boldsymbol{m}\boldsymbol{i}{\boldsymbol{n}}_{\boldsymbol{A}}}\left(\boldsymbol{new}\_\boldsymbol{m}\boldsymbol{a}{\boldsymbol{x}}_{\boldsymbol{A}}\kern0.5em  - \boldsymbol{new}\_\boldsymbol{m}\boldsymbol{i}{\boldsymbol{n}}_{\boldsymbol{A}}\right)+\boldsymbol{new}\_\boldsymbol{m}\boldsymbol{i}{\boldsymbol{n}}_{\boldsymbol{A}} $$

##### Feature selection

**Fig. 3:**
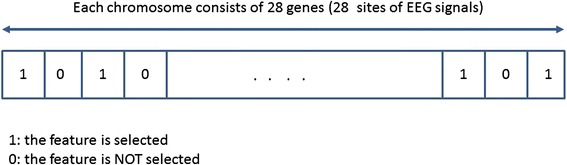
Chromosome representation

Genetic Algorithm (GA): This is a randomized search algorithm used to find an optimal solution for a problem via the process of natural selection in data mining [47]. In GA, “chromosomes” are created randomly to represent the features of the data as *genes*. Each gene is assigned with a string of either 0 or 1, where 0 means the feature is not selected in that particular chromosome whereas 1 means the feature is selected [48]. For example, Fig. 3 represents a chromosome created for EEG data for each band where genes reflect the 28 electrode sites.

Each chromosome represents a point in the search space and a number of chromosomes in the space are called a population. In GA, chromosomes are compared to each other to assess the goodness of each one in solving the problem. This is done by using the *fitness function* that evaluates and assigns a score for each chromosome to select the best ones during the selection phase*.*

Fitness Function: In the fitness function, each chromosome represents features that are selected by GA. Based on the selected features for each chromosome; a dataset is generated and is given to the predictive model (classifier) to evaluate the goodness of the chromosome. Before feeding the classifier with the dataset, the dataset is mapped using Linear Discrimination Analysis (LDA) and then divided into training and testing sets. After that, the training set is fed into the decision tree (classifier) to build a classification model. Finally, the performance of the classifier is evaluated using the testing set to determine how well the classifier performed by computing the *accuracy*. To assign the score for each chromosome, the fitness function is computed using the formula (2).

2 $$ \mathbf{Fitness} = \mathbf{Accuracy} - \mathbf{100}\ \left(\frac{\boldsymbol{Number}\ \boldsymbol{of}\ \boldsymbol{selected}\ \boldsymbol{features}}{\boldsymbol{Total}\ \boldsymbol{number}\ \boldsymbol{of}\ \boldsymbol{feartures}}\right) $$

Based on the formula, the chromosomes which contain fewer features and provide higher detection rate will receive a higher chance of selection for the next population.

Selection Operator (Tournament Selection): Each chromosome in the initial population goes through the same process as described above. Once all the chromosomes are assigned scores, they are then compared with each other in order to select the best ones to reproduce the next population. In this study, the *Binary Tournament Selection* was used [48–51]. In the Binary Tournament Selection algorithm two chromosomes are first selected randomly and based on their fitness function, the one that has a better fitness function is selected and a copy of that chromosome is sent to the next population. The selection algorithm repeats the same process until *n* samples are selected (*n* is number of samples in the initial population). Based on the proposed fitness function, the chromosomes that have higher fitness value will have a greater chance of being selected.

**Fig. 4:**
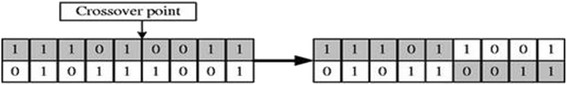
Crossover operator—one point

Crossover Operator (One-point): The selected population during the fitness function needs to be reproduced to create a new generation of chromosomes for the next iteration. The crossover method is used to reproduce new chromosomes for the next population by selecting two parent chromosomes. From these, two new offspring chromosomes are produced by applying the crossover. For example in Fig. 4, parent chromosomes consist of 111010011 and 010111 001, the crossover method randomly chooses loci for exchanging between parents to create offspring with 11101*1001* and 01011*0011* [48–51]*.*

Mutation Operator (Bit Flip): Another method that is employed to produce a new generation of chromosomes is a *Mutation*, which randomly flips a bit in a single chromosome. For example, in the chromosome 111010011, the third locus is flipped to 110010011 [48].

##### Feature mapping

**Fig. 5:**
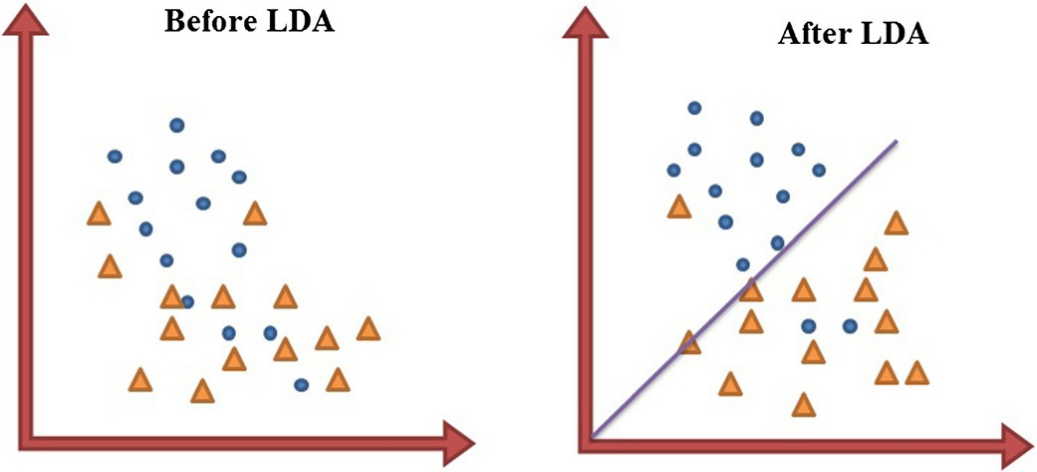
Linear discriminant analysis

Linear Discriminant Analysis (LDA) is a method used in both feature mapping as well as a dimensional reduction and classification. LDA was used as a feature mapping method in order to transfer the original data into a new space where different classes can be discriminated linearly by finding a decision region between the given classes in the newly mapped space that best maximizes the class separability [50]. Figure 5 shows a two dimensional dataset before and after applying LDA. With LDA, the features are mapped into a new feature space in which they are more linearly discriminant compared with the original feature.

LDA faces difficulties in cases of high dimensional data, where the LDA matrices are almost always singular [51]. In our dataset, in some experiments (e.g. Tables 6 & 7); there are more than 110 features while the training dataset consists of less than 100 samples. This indicates that it is not feasible to employ LDA directly on the data. Having observed the low performance of applying mere LDA on our datasets at the initial stage of the study, we decided to first employ GA to reduce the data dimension and then apply LDA to improve the accuracy of final classifier.

Once the four steps of the preprocessing phase were completed successfully, the datasets were ready to be classified by applying the classification model described below.

#### Data modeling

A Decision Tree (DT) was the selected model for this study. Specifically, the C4.5 decision tree model was used. During the training phase, 70 % of the dataset was used to build a classification model that predicts the correct label of the testing set (consisting of the rest (30 %) of the datasets).

#### Data evaluation

The performance of the DT model was evaluated based on counting the test records that were correctly and incorrectly predicted by the DT. The Confusion Matrix provided information that allowed us to determine how well the model performed by computing the *Accuracy* for correct predictions and *Error Rate* (ER) for incorrect predictions, sensitivity, specificity, positive and negative predicted values [52].

**Table:** 

Measurements	Formula
*Sensitivity*	: A/(A+ C)*100
*Specificity*	: D/(D+ B)*100
*Positive Prediction Value (PPV)*	: A/(A+ B)*100
*Negative Prediction Value (NPV)*	: D/(D+ C)*100
*Positive Likelihood Ratio (LR+)*	: Sensitivity/(100-Specificity)
*Negative Likelihood Ratio (LR-)*	: (100-Sensitivity)/Specificity
*Accuracy*	: A + D/(A + B + C + D)*100

### Data mining overview

**Fig. 6:**
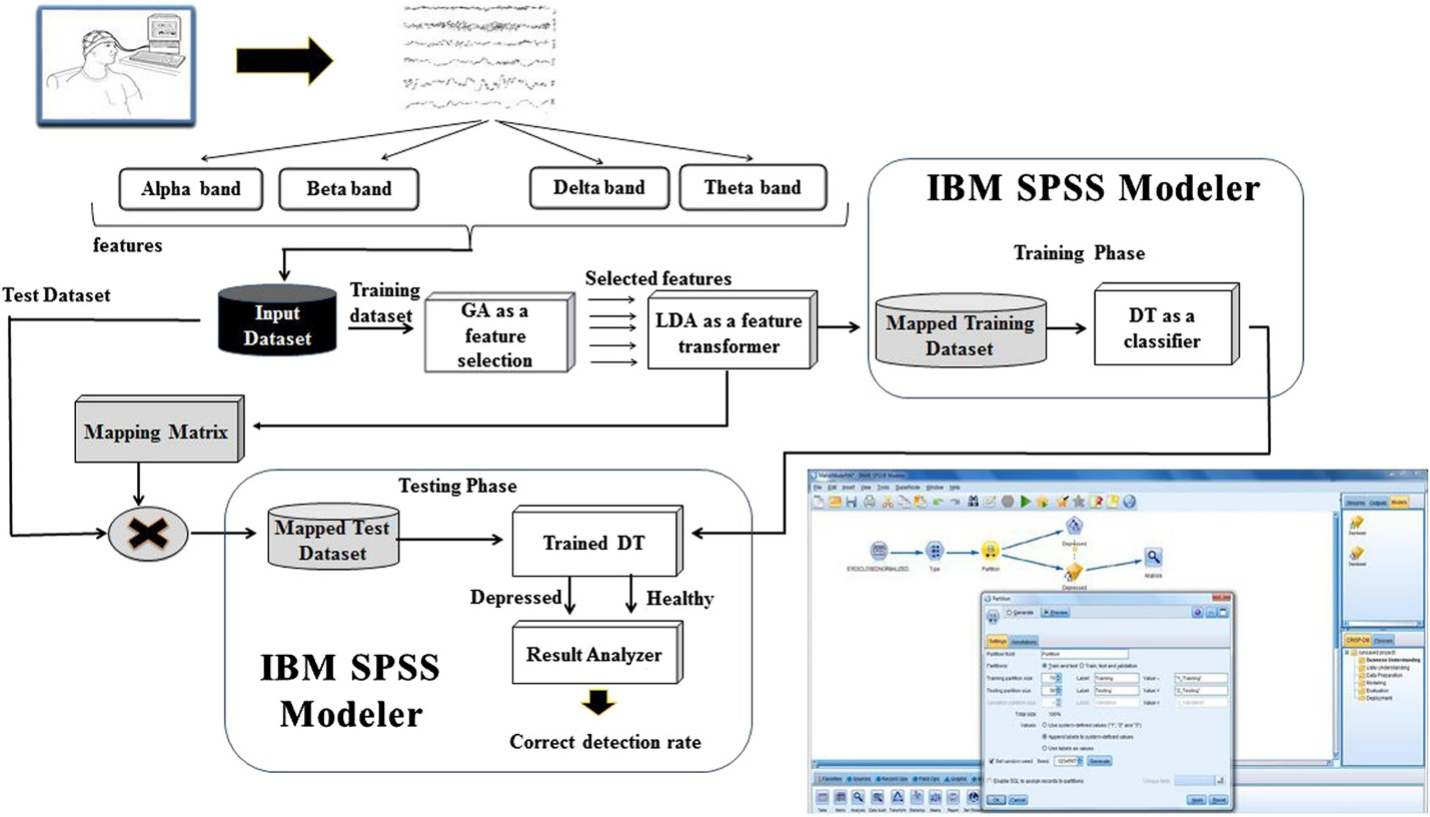
Overview of the data mining methodology applied in this study

Before entering the cleansed and normalized data set into the feature selection phase using the GA, the datasets were divided into training (70 % of dataset) and testing (30 % of dataset) datasets, used for building a predictive model and testing the model’s performance, respectively. As described in Fig. 6, the input dataset contains the training set that is fed to the GA in order to select the best features that discriminate between HVs and MDD patients, and by applying the fitness function *(explained in Feature selection*). The best set of features was then transformed into a new space to linearly separate the data based on the classes by applying LDA. Subsequently, the training set was ready to build the Decision Tree for the predictive model. The features that were selected in the training were also selected in the testing set followed by applying LDA on the testing. Finally, the mapped testing set was then fed into the trained decision tree to test if the model was able to predict MDD patients from HVs and then performance of the model was measured using the accuracy and the error rate of the model (*described in* *Data evaluation* *Evaluation Phase*).

This approach was applied in two sets of analyses. The first was aimed at analyzing each frequency band individually during each of the EO and EC conditions (for each of the mastoid and Cz referenced datasets). Second, data from all bands were analyzed together during EO and EC conditions (for each of the mastoid and Cz referenced datasets). Both analyses were performed using *Matlab* and *IBM SPSS Modeler*.

## Results

The datasets of the four bands (alpha, beta, delta and theta) during the EC and EO conditions were analyzed based on two views to determine the most accurate approach which might be: 1) Each band was analyzed individually during each condition EC and EO for each reference (Mastoid and Cz); and 2) The four bands (alpha, beta, delta and theta) were grouped together to be analyzed as one dataset in each condition EC and EO for Mastoid and Cz references.

Then, the testing dataset was used to validate the predictive model before and after applying GA and LDA. After that, the results were evaluated based on the Sensitivity, Specificity, Positive and Negative Likelihood Rates (LR+ and LR-), Positive and Negative Predictive Values (PPV and NPV), accuracy, and Error rate for depressed and healthy individuals, see sections Mastoid reference—bands analyzed individually, Cz reference—bands analyzed individually, Mastoid reference—bands analyzed together, and Cz reference—bands analyzed together for more details of the analysis. In addition, Section Model evaluation presents the results of the new obtained datasets, and it is used to evaluate the model that consists of the whole dataset that is used through Sections Mastoid reference—bands analyzed individually, Cz reference—bands analyzed individually, Mastoid reference—bands analyzed together, and Cz reference—bands analyzed together.

### Mastoid reference—bands analyzed individually

The results of analyzing each band separately during the EO and EC conditions using a mastoid reference are presented in Table 2. Apart from the delta band, classification error rates were relatively high as evidenced by accuracies ranging from 40–66 %. Low specificities were also noted in non-delta bands (range: 0–54 %). With more than half of the classifiers, sensitivity, specificity, Positive Likelihood Ratio (LR+) and NPV (Negative Predictive Value) rates increased following GA and LDA application but this was not necessarily associated with higher accuracy. Delta was the exceptional individual band classifier when analysed during EO. Although showing less than chance accuracy when analyzed with all candidate features, feature reduction with GA and LDA markedly increased sensitivity, specificity and accuracy rates > 80 %. These results were accompanied by increases and decreases in LR+ and LR-, respectively. As displayed in “Additional file 1: Table S1” the specific scalp sites contributing to these findings were distributed over frontal, central and posterior regions of both hemispheres.

**Table 2:** Results- analysis of the individual bands- mastoid reference

Bands	Status	# of features	Sen. (%)	Spec. (%)	LR+	LR-	PPV (%)	NPV (%)	Acc. (%)	Error depressed	Error healthy
Alpha 8–10.5 Hz	EC	28 Raw Features	64	Inf	Inf	Inf	100	0	64	0	10
		12 (GA + LDA)	71	55	1.29	0.65	67	50	61	6	5
	EO	28 Raw Features	75	63	1.2	0.67	17	90	43	15	1
		15 (GA + LDA)	56	80	0.69	2.22	56	20	43	8	8
Alpha 10.5–13 Hz	EC	28 Raw Features	67	63	1.06	0.9	33	70	46	12	3
		11 (GA + LDA)	67	62	1.08	0.87	56	50	54	8	5
	EO	28 Raw Features	77	53	1.44	0.49	56	70	61	8	3
		10 (GA + LDA)	82	36	2.26	0.28	78	70	75	4	3
Alpha 8–13 Hz	EC	28 Raw Features	64	65	0.98	1.03	39	60	46	11	4
		5 (GA + LDA)	73	59	1.24	0.66	44	70	54	10	3
	EO	28 Raw Features	77	53	1.44	0.49	56	70	61	8	3
		14 (GA + LDA)	100	38	2.67	0	67	100	79	6	0
Beta	EC	28 Raw Features	57	71	0.8	1.5	44	40	43	6	10
		10 (GA + LDA)	64	64	1	1	50	50	50	9	5
	EO	28 Raw Features	59	73	0.81	1.51	56	30	46	7	8
		11 (GA + LDA)	67	63	1.07	0.89	44	60	50	10	4
Delta	EC	28 Raw Features	67	64	1.04	0.93	11	90	39	16	1
		18 (GA + LDA)	56	75	0.75	1.75	50	30	43	9	7
	EO	28 Raw Features	58	69	0.85	1.33	39	50	43	11	5
		10 (GA + LDA)	88	27	3.24	0.16	83	80	82	2	3
Theta	EC	28 Raw Features	64	Inf	Inf	Inf	100	0	64	0	10
		18 (GA + LDA)	79	50	1.57	0.43	61	70	64	7	3
	EO	28 Raw Features	69	58	1.18	0.75	61	50	57	7	5
		10 (GA + LDA)	75	50	1.5	0.5	67	60	64	6	4

### Cz reference—bands analyzed individually

The results of analyzing each band separately during the EC and EO conditions using a Cz reference are presented in Table 3. Although sensitivity, PPV and NPV rates for some of the classifiers reached 100 %, with the exception of the alpha (8–10.5 Hz) band, accuracy rates associated with delta, theta and beta were relatively low (43–64 % over EO/EC conditions). Similarly modest accuracy and sensitivity rates (64 %) were observed with EC and EO alpha band analysis using all candidate features. However, GA and LDA feature extraction processes increased the EC alpha classifier’s sensitivity, PPV, NPV and accuracy rates to 94, 83, 90 and 86 %, respectively. Scalp recordings contributing to these classifications were spread diffusely over frontal, central and parieto-occipital regions (“Additional file 1: Table S2”).

**Table 3:** Results- analysis of the individual bands- Cz reference

Bands	Status	# of features	Sen. (%)	Spec. (%)	LR+	LR-	PPV (%)	NPV (%)	Acc. (%)	Error depressed	Error healthy
Alpha 8–10.5 Hz	EC	28 Raw Features	65	50	1.31	0.69	94	10	64	1	9
		10 (GA + LDA)	94	25	3.75	0.08	83	90	86	3	1
	EO	28 Raw Features	65	50	1.31	0.69	94	10	64	1	9
		15 (GA + LDA)	73	54	1.36	0.58	61	60	61	7	4
Alpha 10.5–13 Hz	EC	28 Raw Features	67	63	1.07	0.89	44	60	50	10	4
		4 (GA + LDA)	64	Inf	Inf	Inf	100	0	64	0	10
	EO	28 Raw Features	64	Inf	Inf	Inf	100	0	64	0	10
		9 (GA + LDA)	74	44	1.66	0.47	78	50	68	4	5
Alpha 8–13 Hz	EC	28 Raw Features	64	Inf	Inf	Inf	100	0	64	0	10
		7 (GA + LDA)	100	57	1.77	0	28	100	54	13	0
	EO	28 Raw Features	64	Inf	Inf	Inf	100	0	64	0	10
		9 (GA + LDA)	100	52	1.91	0	39	100	61	11	0
Beta 8–10.5 Hz	EC	28 Raw Features	58	69	0.85	1.33	39	50	43	10	5
		8 (GA + LDA)	62	67	0.92	1.15	44	50	46	11	5
	EO	28 Raw Features	58	69	0.85	1.33	39	50	43	10	5
		9 (GA + LDA)	75	63	1.2	0.67	17	90	43	15	1
Beta 10.5–13 Hz	EC	28 Raw Features	59	73	0.81	1.51	56	30	46	8	7
		8 (GA + LDA)	100	44	2.25	0	56	100	71	0	8
	EO	28 Raw Features	62	100	0.62	Inf	89	0	57	2	10
		11 (GA + LDA)	74	44	1.68	0.46	78	50	68	4	5
Beta 8–13 Hz	EC	28 Raw Features	64	0	0.64	Inf	100	0	64	0	10
		10 (GA + LDA)	64	0	0.64	Inf	100	0	64	0	10
	EO	28 Raw Features	64	0	0.64	Inf	100	0	64	0	10
		10 (GA + LDA)	83	70	2.77	0.24	83	70	79	3	2
Delta	EC	28 Raw Features	64	0	0.64	Inf	100	0	64	0	10
		9 (GA + LDA)	64	0	0.64	Inf	100	0	64	0	10
	EO	28 Raw Features	64	0	0.64	Inf	100	0	64	0	10
		10 (GA + LDA)	80	61	1.31	0.51	90	22	46	14	1
Theta	EC	28 Raw Features	64	0	0.64	Inf	100	0	64	0	10
		9 (GA + LDA)	100	57	1.77	0	28	100	54	13	0
	EO	28 Raw Features	77	53	1.44	0.49	56	70	61	8	3
		12 (GA + LDA)	64	0	0.64	Inf	100	0	64	0	10

### Mastoid reference—bands analyzed together

Table 4 presents the results of analyzing all of the bands together using either the total (8–13 Hz), or by separating the low (8–10.5 Hz) or high (10.5–13 Hz) alpha band data during EO and EC conditions. Overall, accuracy rates relying on all candidate features were relatively low (56–64 %) but the classification accuracy of MDD patients and HVs significantly increased after feature selection with GA and LDA, regardless of whether alpha total (85–86 %), low (88–89 %) or high alpha band (80–86 %) features were used in the modeling. Of these, the best results were obtained when reduced EC low alpha features were used for classification yielding an accuracy rate approaching 90 %, high sensitivity and specificity rates (89 %), and LR+ (8.09) and LR- (0.12) values that strongly support this model for ruling-in and ruling-out depression. While the “Additional file 1: Tables S3-S5” show that multiple recording regions contributed to these classifiers, left and right parietal and occipital recording sites (where alpha power is typically maximal) did not contribute to these results.

**Table 4:** Mastoid reference results (For around 28 records)

Status	# of features	Sen. (%)	Spec. (%)	LR+	LR-	PPV (%)	NPV (%)	Acc. (%)	Error depressed	Error healthy
Alpha (8–10.5 Hz), Beta, Delta & Theta										
EC	112 Raw Features	69	43	1.21	0.72	53	60	56	8	4
	60 GA + LDA	89	89	8.09	0.12	94	80	89	1	2
EO	112 Raw Features	68	50	1.36	0.64	83	30	64	3	7
	58 GA + LDA	94	75	3.76	0.08	83	90	88	3	1
Alpha (10.5–13 Hz), Beta, Delta & Theta										
EC	112 Raw Features	73	78	3.32	0.35	80	70	75	2	3
	46 GA + LDA	77	100	Inf	0.23	100	70	86	0	3
EO	112 Raw Features	62	71	2.14	0.54	80	50	65	2	5
	42 GA + LDA	80	80	4	0.24	80	80	80	2	2
Alpha (8–13 Hz), Beta, Delta & Theta										
EC	112 Raw Features	62	33	0.93	1.15	76	20	56	4	8
	60 GA + LDA	88	80	4.4	0.15	88	80	85	2	2
EO	112 Raw Features	67	40	1.12	0.83	67	40	57	6	6
	58 GA + LDA	94	75	3.76	0.08	83	90	86	3	1

### Cz reference—bands analyzed together

The results of the analysis of all the bands together using either the total (8–13 Hz), or by separating the low (8–10.5 Hz) and high (10.5–13 Hz) alpha band data during EO and EC conditions are presented in Table 5.

**Table 5:** Cz reference results

Status	# of features	Sen. (%)	Spec. (%)	LR+	LR-	PPV (%)	NPV (%)	Acc. (%)	Error depressed	Error healthy
Alpha (8–10.5 Hz), Beta (8–10.5 Hz), Delta & Theta										
EC	109 Raw Features	55	28	0.76	1.61	32	50	38	13	5
	59 GA + LDA	100	77	4.35	0	84	100	90	3	0
EO	109 Raw Features	55	72	1.96	0.63	32	50	34	13	5
	55 GA + LDA	85	78	3.86	0.19	89	70	83	2	3
Alpha (10.5–13 Hz), Beta (10.5–13 Hz), Delta & Theta										
EC	109 Raw Features	88	53	1.87	0.23	47	90	64	8	1
	50 GA + LDA	100	77	4.35	0	80	100	88	3	0
EO	109 Raw Features	88	53	1.87	0.23	47	90	64	8	1
	57 GA + LDA	90	60	2.25	0.16	60	90	72	6	1
Alpha (8–13 Hz), Beta (8–13 Hz), Delta & Theta										
EC	109 Raw Features	47	14	0.55	3.79	37	20	31	12	8
	59 GA + LDA	94	75	3.76	0.08	84	90	86	3	1
EO	109 Raw Features	75	38	1.21	0.66	32	80	48	13	2
	64 GA + LDA	100	66	2.94	0	74	100	83	5	0

As shown with mastoid-referenced analyses, analyzing all bands together using total raw features of Cz referenced EEG yielded low diagnostic accuracy rates between 31–64 % across EO and EC behavioural states. Similarly, feature extraction with GA and LDA elevated accuracies with total alpha (EC/EO: 86, 83 %), low alpha (EC/EO: 90, 83 %), and high alpha (EC: 88 %) analyses. Further, the more robust classifications were seen in the analysis conducted with reduced low alpha features under the EC condition which also resulted in sensitivity and NPV rates of 100 %, along with moderate rates of specificity (77 %), LR+ (4.35) and LR- (0). The scalp regions contributing to the low alpha classifier were relatively widespread (Additional file 1: Tables S3-S5”).

### Model evaluation

Tables 6 and 7 present the results when newly recorded (unseen) EEG recordings are analyzed using all the bands using mastoid or Cz references, and with low, high or total alpha band features.

**Table 6:** Mastoid reference results on the new unseen 44 records

Status	# of features	Sen. (%)	Spec. (%)	LR+	LR-	PPV (%)	NPV (%)	Acc. (%)	Error depressed	Error healthy
Alpha (8–10.5 Hz), Beta, Delta & Theta										
EC	112 Raw Features	56	51	1.1	0.8	56	51	52	4	17
	60 GA + LDA	78	74	3.02	0.2	78	74	75	2	9
EO	112 Raw Features	22	71	0.7	1.08	22	71	61	7	10
	58 GA + LDA	78	80	3.8	0.2	78	80	80	2	7
Alpha (10.5–13 Hz), Beta, Delta & Theta										
EC	112 Raw Features	56	60	1.3	0.7	56	60	59	4	14
	46 GA + LDA	78	69	2.4	0.3	78	69	70	2	11
EO	112 Raw Features	44	66	1.2	0.8	44	66	61	5	12
	42 GA + LDA	67	74	2.5	0.4	67	74	73	3	9
Alpha (8–13 Hz), Beta, Delta & Theta										
EC	112 Raw Features	33	57	0.7	1.1	33	57	52	6	15
	60 GA + LDA	78	77	3.4	0.2	78	77	77	2	8
EO	112 Raw Features	56	43	0.9	1.03	56	43	45	4	20
	58 GA + LDA	67	74	2.5	0.4	67	74	73	3	9

**Table 7:** Cz Reference Results on the new unseen 44 records

Status	# of features	Sen. (%)	Spec. (%)	LR+	LR-	PPV (%)	NPV (%)	Acc. (%)	Error depressed	Error healthy
Alpha (8–10.5 Hz), Beta (8–10.5 Hz), Delta & Theta										
EC	109 Raw Features	56	43	0.9	1.03	56	43	45	4	20
	59 GA + LDA	67	80	3.3	0.4	67	80	77	3	7
EO	109 Raw Features	11	54	0.2	1.6	11	54	45	8	16
	55 GA + LDA	67	77	2.9	0.4	67	77	75	3	8
Alpha (10.5–13 Hz), Beta (10.5–13 Hz), Delta & Theta										
EC	109 Raw Features	33	66	0.9	1.01	33	66	59	6	12
	50 GA + LDA	78	77	3.4	0.2	78	77	77	2	8
EO	109 Raw Features	56	60	1.3	0.7	56	60	59	4	14
	57 GA + LDA	67	74	2.5	0.4	67	74	73	3	9
Alpha (8–13 Hz), Beta (8–13 Hz), Delta & Theta										
EC	109 Raw Features	33	57	0.7	1.1	33	57	52	6	15
	59 GA + LDA	56	80	2.7	0.5	56	80	75	4	7
EO	109 Raw Features	22	54	0.4	1.4	22	54	48	7	16
	64 GA + LDA	67	80	3.3	0.4	67	80	77	3	7

Although accuracy was frequently below 60 % for mastoid and Cz referenced datasets using all candidate features, classification rates improved to > 70 % using the reduced features derived with GA and LDA. Accuracy reached 75–77 % levels with EC and EO Cz referenced EEG, but the maximal 80 % accuracy was evidenced with EO low alpha analysis, which also yielded sensitivity and specificity rates of 77 % and 80 %, respectively, together with relatively high PPV (78 %) and NPV (80 %) values.

As indicated in Tables 6 and 7, the model shows an acceptable performance on the newly recorded data. The accuracy of the model fluctuates between 70 % and 80 %. For example, based on the Cz reference, EC dataset with all bands (Table 7), the accuracy of the model on 106 raw features is about 60 %, while by applying the proposed method, the accuracy reaches 72.7 % which is a noticeable improvement.

## Discussion

The heterogeneity of symptom profiles and severity among patients with MDD is a major challenge for diagnostic classification. Further, given the reliability problems associated with subjective assessments of clinical phenomena there is an increasing effort to identify more brain-based, objective and reliable classifiers for various psychiatric disorders, including depression. In this paper, classification approaches were performed on EEG signal features (power density in different frequency bands) derived from multiple scalp recording sites, during two states (EO/EC) and analyzed using two reference montages. In Experiment 1, individual bands resulted in relatively high classification errors, regardless of whether or not the complexity and redundancy of signal features was reduced by the genetic algorithm. Exceptions were observed with EO mastoid-referenced delta and EC Cz-referenced total alpha, with the reduced extracted features of each band exhibiting > 80 % accuracy, sensitivity and PPVs. These latter findings are generally supportive of previous group-level comparison studies showing activity of alpha, and to a lesser extent delta oscillations to distinguish depressed and healthy volunteer samples [26].

When analyzing each band separately, classification was similarly less than optimal in Experiment 2 when bands were analyzed together and with the total set of candidate features, but was markedly increased following feature reduction. Regardless of the type of reference (mastoid vs. Cz) or vigilance state (EC vs. EO), most models exhibited low classification errors, with high accuracy and sensitivity values. The electrode sites contributing to these classifiers and the above-mentioned single band delta and alpha classifiers were widely distributed across frontal, temporal and posterior regions in both hemispheres. These data are consistent with functional neuroimaging studies that tend to characterize depression as a dysfunction in a network(s) of discrete, but functionally integrated, cortico-limbic pathways [53], which can be assessed by brain-based algorithms for diagnosis and optimized treatment [54, 56].

In summary, the most accurate decision tree models (accuracies > 80 %) were evaluated with unseen data from 44 participants, including 35 HVs and 9 MDD patients. Correct diagnosis rates of the models were found to be quite accurate. These results generally support the notion that data mining techniques, and especially those involving feature extraction, may yield promising classifiers for the EEG signal processing applications, specifically in cases of MDD and control subjects classification. Improved classification accuracies may possibly be achieved with the addition of other candidate features besides EEG power including EEG coherence and cordance measures, which have been reported to distinguish depressed patients from healthy volunteers, and the EEG antidepressant response (ATR) index, which has predicted treatment response in depressed patients [26, 27].

## Conclusions

In this study we demonstrated that data mining applied to EEG signals may be a useful tool in discriminating between depressed and healthy individuals. Given the questionable reliability of diagnoses based on clinical symptoms, this quantitative methodology may be a useful adjunctive clinical decision support for identifying depression and it supports independent studies confirming the potential clinical utility of computer-aided diagnosis of depression using EEG signals [55].

## Additional file

Additional file 1: Table S1. Feature Selection—Individually Analyzed Mastoid Referenced EEG Bands. **Table S2.** Feature Selection—Individually Analyzed Cz Referenced EEG Bands. **Table S3.** Feature Selection—Combined Analyzed Bands Using the Low (8–10.5) Hz) Alpha EEG Band. **Table S4.** Feature Selection—Combined Analyzed Bands Using the High (10.5–13 Hz) Alpha EEG Band. **Table S5.** Feature Selection—Combined Analyzed Bands Using the Total (8.5–13 Hz) Alpha EEG Band. (DOCX 44 kb)

## Abbreviations

DSM-5: Diagnostic and Statistical Manual of Mental Disorders, 5th edition
DT: Decision tree
EEG: Electroencephalography
FFT: Fast Fourier Transform
fMRI: Functional magnetic resonance imaging
GA: Genetic algorithm
LDA: Linear Discriminant analysis
LR+: Positive Likelihood Ratio
LR-: Negative Likelihood Ratio
MADRS: Montgomery-Asberg Depression Rating Scale
MDD: Major depressive disorder
NPV: Negative Predictive Value
PET: Positron emission tomography
PPV: Positive Predictive Value
SCID: Structured Clinical Interview for DSM
