# Supplementary material for: Data mining EEG signals in depression for their diagnostic value
Source: BMC Med Inform Decis Mak. 2015 Dec 23;15:108. doi: 10.1186/s12911-015-0227-6 (PMC4690290; doi:10.1186/s12911-015-0227-6)
Supplement: Additional file 1: Table S1. — Feature Selection—Individually Analyzed Mastoid Referenced EEG Bands. Table S2. Feature Selection—Individually Analyzed Cz Referenced EEG Bands. Table S3. Feature Selection—Combined Analyzed Bands Using the Low (8–10.5) Hz) Alpha EEG Band. Table S4. Feature Selection—Combined Analyzed Bands Using the High (10.5–13 Hz) Alpha EEG Band. Table S5. Feature Selection—Combined Analyzed Bands Using the Total (8.5–13 Hz) Alpha EEG Band. (DOCX 44 kb) [file 12911_2015_227_MOESM1_ESM.docx]

**Additional file 1**

***Table S1. Feature Selection – Individually Analyzed Mastoid Referenced EEG Bands***

The table lists the four bands with their selected features (electrode sites) based on application of the Genetic Algorithm (GA) to each band processed individually with EC-mastoid (□) and EO-mastoid (○) referenced EEG features.

| **References (Brain Sites)** | **Alpha**  **8-10.5 Hz** | **Alpha**  **10.5-13Hz** | **Alpha**  **8-13Hz** | **Beta** | **Delta** | **Theta** |
| --- | --- | --- | --- | --- | --- | --- |
| **Fp1** | □○ |  | ○ | □○ | □ | □ |
| **Fp2** | □○ | ○ |  |  | □ |  |
| **F3** | □ | □ | ○ | ○ | □○ | □ |
| **F4** |  | ○ | ○ | □○ | □○ | ○ |
| **C3** |  |  |  | □○ | ○ | □○ |
| **C4** | ○ | □ |  | □○ | □ |  |
| **P3** | □○ |  |  | ○ | □ | ○ |
| **P4** | □ |  |  |  | ○ |  |
| **O1** |  |  | ○ | □○ | □ |  |
| **O2** | ○ | □ | ○ | □ | ○ | □ |
| **F7** | □○ | □○ |  |  |  |  |
| **F8** | □○ |  | ○ | □ |  |  |
| **T7** |  |  |  | ○ |  | □ |
| **T8** | ○ | □○ | □ | ○ | □ | □ |
| **P7** | ○ | □ |  |  |  | □ |
| **P8** | □○ | ○ | □○ | □ | □ | □○ |
| **Fz** |  | ○ |  |  | □ | □ |
| **Cz** | □ | ○ | □ |  |  |  |
| **Pz** | ○ | □○ | ○ | □ | □○ | □○ |
| **Oz** |  |  | □ | ○ | □○ | ○ |
| **Fc1** | □○ |  | ○ |  | ○ | □ |
| **Fc2** |  | ○ | ○ |  | □ | ○ |
| **Cp1** | ○ | □ | ○ |  | □ |  |
| **Cp2** | □○ | □○ | ○ |  |  | □ |
| **Fc5** |  |  |  | ○ |  |  |
| **Fc6** |  | □ | □○ | □ | □○ | □○ |
| **Cp5** |  | □ |  |  | □ | ○ |
| **Cp6** | □○ |  | ○ |  | ○ | ○ |

***Table S2. Feature Selection – Individually Analyzed Cz Referenced EEG Bands***

The table lists the four bands with their selected features (electrode sites) based on application of the Genetic Algorithm (GA) to for each band processed individually with EC-Cz (⯎) and EO-Cz (⯏) referenced EEG features.

| **References (Brain Sites)** | **Alpha**  **8-10.5 Hz** | **Alpha**  **10.5-13Hz** | **Alpha**  **8-13Hz** | **Beta**  **8-10.5 Hz** | **Beta**  **10.5-13Hz** | **Beta**  **8-13Hz** | **Delta** | **Theta** |
| --- | --- | --- | --- | --- | --- | --- | --- | --- |
| **Fp1** | ⯎⯏ |  | ⯎ |  | ⯏ |  | ⯏ | ⯎ |
| **Fp2** | ⯏ |  | ⯎ | ⯎⯏ |  |  | ⯎⯏ | ⯎⯏ |
| **F3** | ⯏ | ⯎⯏ | ⯎⯏ | ⯏ |  | ⯎⯏ | ⯏ | ⯎⯏ |
| **F4** | ⯎⯏ | ⯎⯏ | ⯎ | ⯏ | ⯏ | ⯏ | ⯎⯏ | ⯎⯏ |
| **C3** | ⯎⯏ | ⯏ | ⯏ | ⯎ |  | ⯎ | ⯏ |  |
| **C4** |  |  |  |  | ⯎⯏ | ⯏ |  | ⯎ |
| **P3** |  |  |  | ⯏ | ⯏ | ⯏ | ⯎⯏ |  |
| **P4** |  | ⯏ |  | ⯎ |  | ⯏ |  |  |
| **O1** | ⯏ |  |  | ⯏ | ⯏ |  | ⯎ |  |
| **O2** | ⯎ |  |  | ⯏ |  |  | ⯏ | ⯏ |
| **F7** |  |  |  |  | ⯏ | ⯏ | ⯎ |  |
| **F8** |  |  |  | ⯎⯏ |  |  | ⯎ | ⯎ |
| **T7** | ⯎⯏ |  | ⯏ | ⯎ | ⯏ |  | ⯏ |  |
| **T8** |  |  |  |  | ⯎ | ⯎ | ⯎ |  |
| **P7** | ⯎⯏ | ⯏ |  | ⯎ |  | ⯎ | ⯎ | ⯏ |
| **P8** | ⯎ | ⯏ |  | ⯎ |  | ⯎ |  | ⯎ |
| **Fz** | ⯏ |  | ⯏ |  |  |  |  | ⯏ |
| **Cz** |  | ⯏ | ⯏ |  |  |  |  |  |
| **Pz** | ⯏ | ⯏ | ⯎ |  | ⯎⯏ | ⯏ |  | ⯏ |
| **Oz** | ⯎⯏ | ⯏ | ⯏ | ⯏ |  | ⯎ |  |  |
| **Fc1** |  |  | ⯏ |  | ⯎ |  | ⯏ |  |
| **Fc2** | ⯎⯏ |  | ⯎ |  | ⯎⯏ | ⯎⯏ |  | ⯏ |
| **Cp1** |  |  |  |  |  | ⯏ |  | ⯏ |
| **Cp2** | ⯏ | ⯎ | ⯏ | ⯏ |  | ⯎ | ⯎⯏ | ⯏ |
| **Fc5** |  |  |  | ⯎ |  | ⯎ |  | ⯎ |
| **Fc6** |  |  |  |  | ⯎ | ⯎ |  | ⯏ |
| **Cp5** | ⯏ | ⯎ |  |  | ⯎⯏ | ⯏ |  |  |
| **Cp6** | ⯎⯏ |  | ⯎⯏ |  | ⯎⯏ |  |  | ⯎⯏ |

***Table S3. Feature Selection - Combined Analyzed Bands Using the Low (8-10.5) Hz) Alpha EEG Band***

The table lists the four bands with their selected features (electrode sites) based on application of the Genetic Algorithm (GA) to bands processed together with EC-mastoid (□), EO-mastoid (○), EC-Cz (⯎) and EO-Cz (⯏) referenced EEG features.

| **References (Brain Sites)** | **Low Alpha** | **Beta** | **Delta** | **Theta** |
| --- | --- | --- | --- | --- |
| **Fp1** | ⯎⯏ | □⯎⯏ | ○ | ○ |
| **Fp2** | □⯎⯏ | ⯏ | ⯎⯏ | □⯏ |
| **F3** | □○⯎⯏ | □○ | ⯏ | ⯎ |
| **F4** | ○ | □○⯏ | □⯎ | ○ |
| **C3** | □○⯎ | □⯎⯏ | ⯎ | ○⯎ |
| **C4** | □ | □⯎ | □⯎⯏ | ⯏ |
| **P3** | ○⯎⯏ | ⯎⯏ | ○⯏ | □⯏ |
| **P4** | ⯎ | □○⯎ | □○⯏ | □⯎⯏ |
| **O1** | ○⯏ | ○⯎ | ○⯎ | ○⯎ |
| **O2** | ⯎ | □○⯎⯏ | □○⯏ | □○⯏ |
| **F7** | □○ | ⯎ | ○⯎ | ⯎ |
| **F8** | ⯎ | □○⯎ | □○⯎⯏ | ○⯎ |
| **T7** | □⯎⯏ | □○⯏ | ⯏ | □⯎⯏ |
| **T8** | □⯏ | □○⯎ | ⯎⯏ | □⯎⯏ |
| **P7** | ⯎ | □⯎ | ○⯏ |  |
| **P8** | □ | □⯎⯏ | ○ | □ |
| **Fz** | □○⯎⯏ | ○⯎⯏ | □○⯏ | ○⯎⯏ |
| **Cz** | □⯏ | □ | ○ | ○ |
| **Pz** | ○⯎⯏ | ⯎⯏ | □⯎ | □○⯏ |
| **Oz** | ○⯏ | □⯎ | □○⯏ | □ |
| **Fc1** | □○⯎⯏ | □○ | □ | □○⯏ |
| **Fc2** | □○⯎ | ○⯎ | ⯎⯏ | □⯎ |
| **Cp1** | ○ | □○⯏ | ○⯎⯏ | □⯏ |
| **Cp2** | ○⯎ | ○ | ○⯎⯏ | □○⯎⯏ |
| **Fc5** | □○ | □ | □ |  |
| **Fc6** | ○⯎ | ○⯏ | □⯎⯏ | □ |
| **Cp5** | □⯏ | □⯎ | ○ | □ |
| **Cp6** | □○⯎ | ○⯏ |  | □○⯎⯏ |

***Table S4. Feature Selection - Combined Analyzed Bands Using the High (10.5-13 Hz) Alpha EEG Band***

The table lists the four bands with their selected features (electrode sites) based on application of the Genetic Algorithm (GA) to bands processed together with EC-mastoid (□), EO-mastoid (○), EC-Cz (⯎) and EO-Cz (⯏) referenced EEG.

| **References (Brain Sites)** | **High Alpha** | **Beta** | **Delta** | **Theta** |
| --- | --- | --- | --- | --- |
| **Fp1** | ○⯏ | □○⯎ | ⯎ | □⯎ |
| **Fp2** | ○⯏ | □○⯏ | ⯎ | □⯎⯏ |
| **F3** | ○⯏ | □⯎ | ⯎⯏ | □○⯏ |
| **F4** | □○⯎ | ○⯎⯏ |  |  |
| **C3** | □⯎ | □○⯏ | ⯏ | □○⯏ |
| **C4** | ⯎ | ⯎ | ○⯎⯏ | □⯎⯏ |
| **P3** | ⯎ | ○⯏ | ⯏ | ⯎⯏ |
| **P4** |  |  | □○ | □⯎ |
| **O1** | □ | ○⯎⯏ | □⯎ | ○⯏ |
| **O2** |  | ○⯎⯏ |  | ○ |
| **F7** | □ | ○⯎ | ⯏ | ⯏ |
| **F8** | □○⯏ | □○ | ⯏ | □ |
| **T7** | ○⯎⯏ | ⯏ | ○⯎⯏ | ⯏ |
| **T8** | ⯏ | ○⯎ | ⯎ |  |
| **P7** | □○⯏⯎ | □⯏ | ⯏ | ⯏ |
| **P8** | □⯎⯏ | ⯏ | □⯎ | ⯏ |
| **Fz** | □⯎ | □⯏ |  | □⯎⯏ |
| **Cz** | □○⯏ | ○ | ○ | □ |
| **Pz** | ⯎ | ○ | ⯎ |  |
| **Oz** | ○ | ⯏ | ○⯎⯏ | □ |
| **Fc1** | □○⯎⯏ |  | □○⯎ | □⯎⯏ |
| **Fc2** | □⯎ | ○ | ⯏ | □ |
| **Cp1** | □○⯏ | ○⯎⯏ | ⯎⯏ | ⯎⯏ |
| **Cp2** | ○⯏ | ⯏ | □⯎ | □○⯎ |
| **Fc5** | □⯎ |  | □⯎ |  |
| **Fc6** | □○ | □⯎⯏ |  | □○⯏ |
| **Cp5** | □⯎⯏ | ○ | □⯎ | ⯏ |
| **Cp6** | ○⯎⯏ | ⯎⯏ | □○⯏ | □⯎⯏ |

***Table S5. Feature Selection - Combined Analyzed Bands Using the Total (8.5-13 Hz) Alpha EEG Band***

The table lists the four bands with their selected features (electrode sites) based on application of the Genetic Algorithm (GA) to bands processed together with EC-mastoid (□), EO-mastoid (○), EC-Cz (⯎) and EO-Cz (⯏) referenced EEG.

| **References (Brain Sites)** | **Total Alpha** | **Beta** | **Delta** | **Theta** |
| --- | --- | --- | --- | --- |
| **Fp1** | □⯎⯏ | ○⯎ | □○⯏ |  |
| **Fp2** | ○⯎ | □⯎⯏ | □⯎⯏ | □⯎⯏ |
| **F3** | ○⯏ | □○⯎ | ⯎⯏ | ⯎ |
| **F4** |  | □○ | □⯎⯏ | □⯏ |
| **C3** | □⯏ | □⯏ | □⯎ | ○⯏ |
| **C4** | □○⯏ | ○⯏ | □⯎⯏ | ⯎ |
| **P3** | □○ | ○⯏ | ⯏ | ○⯎⯏ |
| **P4** | □⯎⯏ | □○⯎⯏ | ○⯏ | □⯎⯏ |
| **O1** | ○⯏ | □○⯎ | □○⯎⯏ | ⯎ |
| **O2** | □○⯏ | ○⯎⯏ | □⯎ | □⯎ |
| **F7** | ⯎⯏ | ○⯎⯏ | □⯎ | ⯎⯏ |
| **F8** | □○ | □○⯏ | ⯎⯏ | □○⯎ |
| **T7** | ⯏ | ○⯏ | □○⯏ | ⯎⯏ |
| **T8** | ⯏ | ○ |  | ⯎ |
| **P7** | ○⯎⯏ |  | □○ | ⯎⯏ |
| **P8** | ○⯏ | ⯎ | ○ | ○⯏ |
| **Fz** | □⯏ | □○⯎ | □○⯎ | ⯎ |
| **Cz** |  |  | ○ | □○ |
| **Pz** | □ | □⯏ | □ | □○⯎ |
| **Oz** | □○⯎ | ○⯏ | ⯏ | ⯏ |
| **Fc1** | ○⯎ | ○⯎⯏ | □⯏ | ⯎⯏ |
| **Fc2** | □○⯎⯏ | ⯎ | □○⯎⯏ | ○⯎⯏ |
| **Cp1** | □⯎ | □○ | □○⯏ | ⯎⯏ |
| **Cp2** | ⯏ |  | □⯎ | ⯏ |
| **Fc5** | □⯏ | ○ | ○⯎⯏ | □⯎ |
| **Fc6** | □○⯎ | ○⯎ | ⯏ | ⯎ |
| **Cp5** | ⯏ | □○⯏ | □⯎ | ⯎⯏ |
| **Cp6** | □⯏ | □⯎⯏ | ○⯎⯏ | □⯎ |
